# Does axial length drive retinal displacement after repair of rhegmatogenous retinal detachment following phacoemulsification

**DOI:** 10.1186/s12886-026-04867-w

**Published:** 2026-04-30

**Authors:** Serkan Güler, Murat Karapapak, Ece Özal, Serhat Ermiş, Sadık Altan Özal

**Affiliations:** 1 Department of Ophthalmology Başakşehir Çam and Sakura City Hospital, University of Health Sciences, Istanbul, Türkiye; 2 Department of Ophthalmology, Çorlu State Hospital, Tekirdağ, Türkiye

**Keywords:** Rhegmatogenous retinal detachment, Phacoemulsification, Pars plana vitrectomy, Silicone oil, C₃F₈ gas, Fundus autofluorescence, Retinal displacement, Retinal vessel printings, Axial length, Metamorphopsia

## Abstract

**Purpose:**

To evaluate the incidence and direction of postoperative retinal displacement after pars plana vitrectomy(PPV) for macula-off rhegmatogenous retinal detachment(RRD) developing after phacoemulsification, and to assess associations with pre-phacoemulsification axial length(AL) and postoperative metamorphopsia.

**Methods:**

This retrospective study included 32 eyes with post-phacoemulsification, macula-off pseudophakic RRD treated with primary 25-gauge PPV using either 14% perfluoropropane(C₃F₈) gas or 5,000-cSt silicone oil(SiO) tamponade.Tamponade was preferentially selected by break location: superior breaks received C₃F₈, whereas inferior breaks(4 to 8 o’clock) received SiO.Perfluorocarbon liquid–assisted drainage through the break was performed without retinotomy or internal limiting membrane peeling.Retinal displacement was evaluated on fundus autofluorescence obtained after tamponade clearance, specifically 1 month after SiO removal and 1 month after complete C₃F₈ gas resorption and was defined by retinal vessel printings ≥ 2 optic disc diameters.Metamorphopsia was quantified using M-CHARTS at months 1, 3, and 6.

**Results:**

SiO was used in 19 eyes(59.4%) and C₃F₈ in 13(40.6%).Retinal displacement occurred in 7/19 eyes(36.8%) in the SiO group and 8/13(61.5%) in the C₃F₈ group(*p* = 0.169).Displacement direction differed significantly: SiO showed superior(21.1%) and inferior(15.8%) displacement, whereas C₃F₈ showed exclusively inferior displacement (61.5%)(*p* = 0.016).AL did not differ by displacement status within either group(SiO *p* = 0.922; C₃F₈ *p* = 0.428) and showed no correlation with M-CHARTS change scores at any interval (all *p* ≥ 0.283).M-CHARTS scores (vertical, horizontal, and mean) were comparable between groups at months 1, 3, and 6 (all *p* > 0.05).

**Conclusion:**

In macula-off RRD developing after phacoemulsification, postoperative retinal displacement was common and showed tamponade-specific directionality, uniformly inferior with C₃F₈ and more heterogeneous with SiO.Within this cohort’s AL range, AL was not associated with displacement or metamorphopsia.

**Clinical trial registration:**

Not applicable.

## Introduction

Phacoemulsification is a widely practised surgical procedure worldwide, but it can still lead to serious complications such as rhegmatogenous retinal detachment (RRD) [1, 2]. In a large-scale, systematic review and meta-analysis, the pooled incidence of post-cataract RRD was found to be 0.66 events per 100 patients [1]. Contemporary reviews also report a 10-years cumulative pseudophakic RRD incidence of between 0.36% and 2.9% after phacoemulsification [3, 4]. Risk tends to cluster in higher-risk phenotypes, particularly in individuals with myopia-related biometric factors, such as a longer axial length(AL) [5].

Retinal displacement is being reported at significant rates following pars plana vitrectomy(PPV) surgery, typically in the single digits up to the 60% range, across fundus autofluorescence(FAF)-based series [6]. Functional symptoms remain common after PPV for RRD; a recent study found metamorphopsia in 51.4% at 1 month and 29.9% at 6 months [7]. Importantly, a recent cohort study of patients with retinal detachment reported displacement in 28.2% of cases after surgery. Displacement was found to be strongly associated with postoperative metamorphopsia, occurring more frequently in eyes that had undergone gas tamponade [8].

Although recent studies have suggested that an increase in AL may increase the risk of postoperative retinal displacement [8], evidence confirming this relationship consistently across different clinical scenarios remains limited. In this study, we evaluated postoperative retinal displacement and metamorphopsia in pseudophakic patients who developed RRD following phacoemulsification at our institution. Furthermore, by examining the potential impact of preoperative AL on the occurrence of displacement and metamorphopsia, our aim was to contribute to the existing literature on the relationship between biometric factors and postoperative malapposition patterns, as well as functional outcomes.

## Methods

This retrospective, observational study was conducted at the Department of Ophthalmology, Başakşehir Çam and Sakura City Hospital, Istanbul, Türkiye (ethics committee approval number: KAEK/14.01.2026.11). We reviewed patients who underwent cataract surgery (phacoemulsification) between May 2020 and January 2026 and subsequently developed RRD requiring PPV at our institution. The study conducted in accordance with the Declaration of Helsinki, adhered to established ethical principles.

### Participants

Patients aged 45–80 years who underwent phacoemulsification and subsequently developed RRD in the operated eye were included in the study. Before cataract surgery, patients routinely underwent a dilated fundus examination, paying particular attention to the peripheral retina in eyes considered at risk of retinal detachment. Lesions were documented and managed as needed.AL values were extracted directly from the pre-phacoemulsification biometry records of the study eye. All measurements had been obtained preoperatively using the same optical biometry device, the Tomey OA-2000 Optical Biometer (Tomey Corporation, Nagoya, Japan), according to the routine institutional protocol.A history of other intraocular surgery in the study eye was an exclusion criterion. Eyes with proliferative vitreoretinopathy grade C or higher [9], eyes with concomitant macular pathology and vascular retinal diseases were also excluded. Macula-off detachments were included based on the medical records, with macula-on cases excluded. The extent of foveal involvement was classified preoperatively according to the Klaas et al. [10] method in macula-off eyes. The displacement analysis included only eyes with high-quality FAF images. FAF images obtained one month after SiO removal were evaluated in the SiO tamponade group. As silicone oil is usually removed at around two months postoperatively, FAF assessment in this group was generally performed at approximately three months postoperatively.In the gas tamponade group, FAF images acquired one month after complete C₃F₈ resorption were analysed. Complete resorption of C₃F₈ generally occurred after around 1.5 months, so FAF assessment was performed approximately 2.5 months after primary surgery.

### Surgical procedure

All patients underwent primary RRD repair with PPV under general anaesthesia using a standard three-port, 25-gauge setup with the Alcon Constellation Vision System (Alcon Laboratories, Inc., Fort Worth, TX, USA). The posterior hyaloid was identified and completely detached in all cases, followed by meticulous shaving of the vitreous base to the greatest extent possible. Perfluorocarbon liquid was used in every eye to stabilise the retina and facilitate reattachment. Once the retina had been reattached, all identified breaks were treated with circumferential endolaser photocoagulation around the tears. As these were first-time detachment surgeries, internal limiting membrane peeling was not performed. Subretinal fluid was actively drained from the tear site. No patient underwent retinotomy.

Intraocular tamponade was selected at the end of surgery according to the surgeon’s intraoperative assessment and the overall case characteristics. In our practice, eyes with superior retinal breaks (from 8 to 12 to 4 o’clock) were preferentially tamponaded with 14% perfluoropropane (C₃F₈) gas, whereas eyes with inferior retinal breaks (from 4 to 8 o’clock) were preferentially tamponaded with 5,000-cSt SiO.Immediately after surgery, all patients were placed in a face-down position and instructed to maintain this position during the early postoperative period. In the SiO group, SiO removal was performed at postoperative month 2 if the retina remained attached.

### Patient evaluation

Patient records were reviewed retrospectively using the hospital’s digital medical record system. Demographic data and key clinical variables were extracted. At postoperative week 1 and months 1, 3 and 6, patients underwent examinations, including assessment of best-corrected visual acuity(BCVA) measured in logMAR units, Goldmann applanation tonometry and slit-lamp biomicroscopy. Complications were recorded throughout follow-up. Swept-source optical coherence tomography (SS-OCT) (Triton, Topcon, Japan) was used to assess the macular region and exclude concomitant macular pathology. Displacement assessment was performed using FAF imaging obtained with the Canon CX-1 fundus camera (Canon Inc., Tokyo, Japan), and hyperautofluorescent retinal vessel printings were identified. Retinal displacement was considered present when vessel printings were detected at a distance of at least two optic disc diameters from the corresponding retinal vessels. Postoperative metamorphopsia was evaluated in non-dilated eyes using M-CHARTS™ (Inami & Co., Ltd., Tokyo, Japan) under routine room lighting with near correction.

### Statistical analysis

The statistical analyses in this study were performed using the NCSS (Number Cruncher Statistical System) 2007 statistical software package (Utah, USA). Descriptive statistical methods (mean, standard deviation, median and interquartile range) were used to evaluate the data, and the Shapiro–Wilk normality test was used to examine the distribution of the variables. For variables showing a normal distribution, a paired one-way analysis of variance was used for time comparisons and a Newman-Keuls multiple comparison test for subgroup comparisons. The independent t-test was used for comparisons of two groups, the Friedman test for time comparisons of non-normal distributed variables, the Dunn’s multiple comparison test for subgroup comparisons, the Mann-Whitney U test for comparisons of two groups, the chi-square test and Fisher’s exact test for comparisons of qualitative data and the Pearson correlation test to determine relationships between variables. All results were evaluated at a significance level of *p* < 0.05.

## Results

Between May 2020 and January 2026, a total of 15,328 phacoemulsification procedures were performed in our clinic. Screening identified 35 eyes that developed RRD after phacoemulsification and subsequently underwent PPV.Of these, 3 eyes were excluded because of missing pre-phacoemulsification axial length data in 1 eye and inadequate fundus autofluorescence image quality in 2 eyes.Following application of the predefined exclusion criteria, a total of 32 eyes from 32 patients were included in the analysis (PPV–SiO, *n* = 19; PPV– C₃F₈ gas, *n* = 13).All 32 eyes completed the scheduled visits and assessments.The groups were comparable in terms of age (61.0 ± 9.2 vs. 59.8 ± 8.6 years, *p* = 0.706), sex distribution (female: 57.9% vs. 84.6%, *p* = 0.109) and laterality (right eye: 57.9% vs. 53.8%, *p* = 0.821). There was no significant difference in the distribution of foveal involvement categories between the two groups (*p* = 0.465). Patient demographics and baseline clinical characteristics are detailed in Table 1.

**Table 1:** Baseline demographic, clinical, and surgical characteristics of the study groups

Age		PPV-SiO		PPV-Gas		*p*
	Mean ± SD	61 ± 9,2		59,77 ± 8,64		0,706*
Gender	Female	11	57,89%	11	84,62%	0,109+
	Male	8	42,11%	2	15,38%	
Side	Right	11	57,89%	7	53,85%	0,821+
	Left	8	42,11%	6	46,15%	
Time Between Vision Loss And Surgery (Days)	Mean ± SD	14,11 ± 5,39		11,69 ± 8,19		0,570†
	Median (IQR)	14 (11–18)		16 (2–17)		
Presence Of High Myopia	No	16	84,21%	12	92,31%	0,496+
	Yes	3	15,79%	1	7,69%	
Axial Length (mm)	Mean ± SD	24,39 ± 1,05		24,27 ± 1,38		0,782*
Foveal İnvolvement	incomplete perifoveal detachment	0	0,00%	1	7,69%	0,465+
	incomplete parafoveal detchment	1	5,26%	2	15,38%	
	complete foveal detachment	3	15,79%	1	7,69%	
	complete parafoveal detachment	4	21,05%	4	30,77%	
	complete perifoveal detachment	11	57,89%	5	38,46%	
Number Of Retinal Break	Mean ± SD	1,74 ± 0,99		1,38 ± 0,77		0,290+
Duration Of Operation (min)	Mean ± SD	95,79 ± 26,42		54,69 ± 32,66		**0**,**001**†
	Median (IQR)	95 (80–110)		60 (16,5–80)		

*Independent *t* test †Mann–Whitney *U* test + Chi-square test

p <0.05 was considered statistically significant

PPV: pars plana vitrectomy ; SD: standard deviation ; IQR: interquartile range

BCVA was comparable between groups preoperatively (median 1.8 vs. 1.3 logMAR, Mann–Whitney U test *p* = 0.622) and at postoperative month 1 (Mann–Whitney U test *p* = 0.099), but was significantly better in the PPV–gas group at months 3 and 6 (median 0.22 vs. 0.52 logMAR, Mann–Whitney U test *p* = 0.037; and 0.20 vs. 0.39 logMAR, Mann–Whitney U test *p* = 0.025, respectively). In both groups, BCVA improved significantly over time (Friedman *p* = 0.0001 for each), with post hoc comparisons indicating sustained improvement from baseline across follow-up visits. Intraocular pressure did not differ between groups at baseline and at postoperative week 1 and months 1, 3, and 6 (all *p* > 0.05; baseline *p* = 0.827, week 1 *p* = 0.469, month 1 *p* = 0.996, month 3 *p* = 0.384, month 6 *p* = 0.094).

The rates of macular epiretinal membrane and cystoid macular oedema were comparable between the PPV–SiO and PPV–gas groups at postoperative months 1, 3 and 6 (all *p* > 0.05). Foveal ellipsoid layer disruption was more frequent in the PPV–SiO group at month 1 (47.4% vs. 7.7%, *p* = 0.020). However, no significant differences were observed between the groups at months 3 and 6 (both *p* = 0.185). Within-group longitudinal comparisons showed no significant changes in any OCT finding over time (McNemar tests, all *p* ≥ 0.125).

Postoperative metamorphopsia scores assessed using M-CHARTS™ (vertical, horizontal and mean) were comparable between the PPV–SiO and PPV-gas groups at months 1, 3 and 6, with no significant change observed within either group over time. Detailed data are presented in Table 2.

**Table 2:** Postoperative M-CHARTS™ metamorphopsia scores

M-CHART				PPV-SiO	PPV-Gas	*p*†
Metamorphopsia vertical	Month 1		Mean ± SD	0,31 ± 0,22	0,20 ± 0,21	0,146
			Median (IQR)	0,30 (0,2 − 0,4)	0,20 (0–0,3)	
	Month 3		Mean ± SD	0,28 ± 0,20	0,25 ± 0,32	0,489
			Median (IQR)	0,30 (0,2 − 0,4)	0,20 (0–0,4)	
	Month 6		Mean ± SD	0,32 ± 0,24	0,22 ± 0,18	0,153
			Median (IQR)	0,30 (0,2 − 0,4)	0,20 (0,1 − 0,3)	
	p‡			0,212	0,674	
Metamorphopsia horizontal	Month 1		Mean ± SD	0,23 ± 0,22	0,18 ± 0,18	0,496
			Median (IQR)	0,20 (0–0,5)	0,20 (0–0,25)	
	Month 3		Mean ± SD	0,24 ± 0,25	0,22 ± 0,31	0,779
			Median (IQR)	0,20 (0–0,3)	0,20 (0–0,3)	
	Month 6		Mean ± SD	0,26 ± 0,25	0,22 ± 0,32	0,302
			Median (IQR)	0,30 (0–0,4)	0,20 (0–0,25)	
	p‡			0,651	0,674	
Metamorphopsia mean	Month 1		Mean ± SD	0,27 ± 0,20	0,19 ± 0,18	0,252
			Median (IQR)	0,3 (0,1 − 0,4)	0,20 (0–0,3)	
	Month 3		Mean ± SD	0,26 ± 0,20	0,27 ± 0,41	0,460
			Median (IQR)	0,25 (0,1 − 0,35)	0,15 (0–0,35)	
	Month 6		Mean ± SD	0,29 ± 0,23	0,22 ± 0,23	0,254
			Median (IQR)	0,30 (0,1 − 0,4)	0,20 (0,05 − 0,28)	
		p‡		0,516	0,499	

†Mann-Whitney U test ‡Friedman test

p <0.05 was considered statistically significant

PPV: pars plana vitrectomy;  SD: standard deviation; IQR: interquartile range

**Fig. 1:**
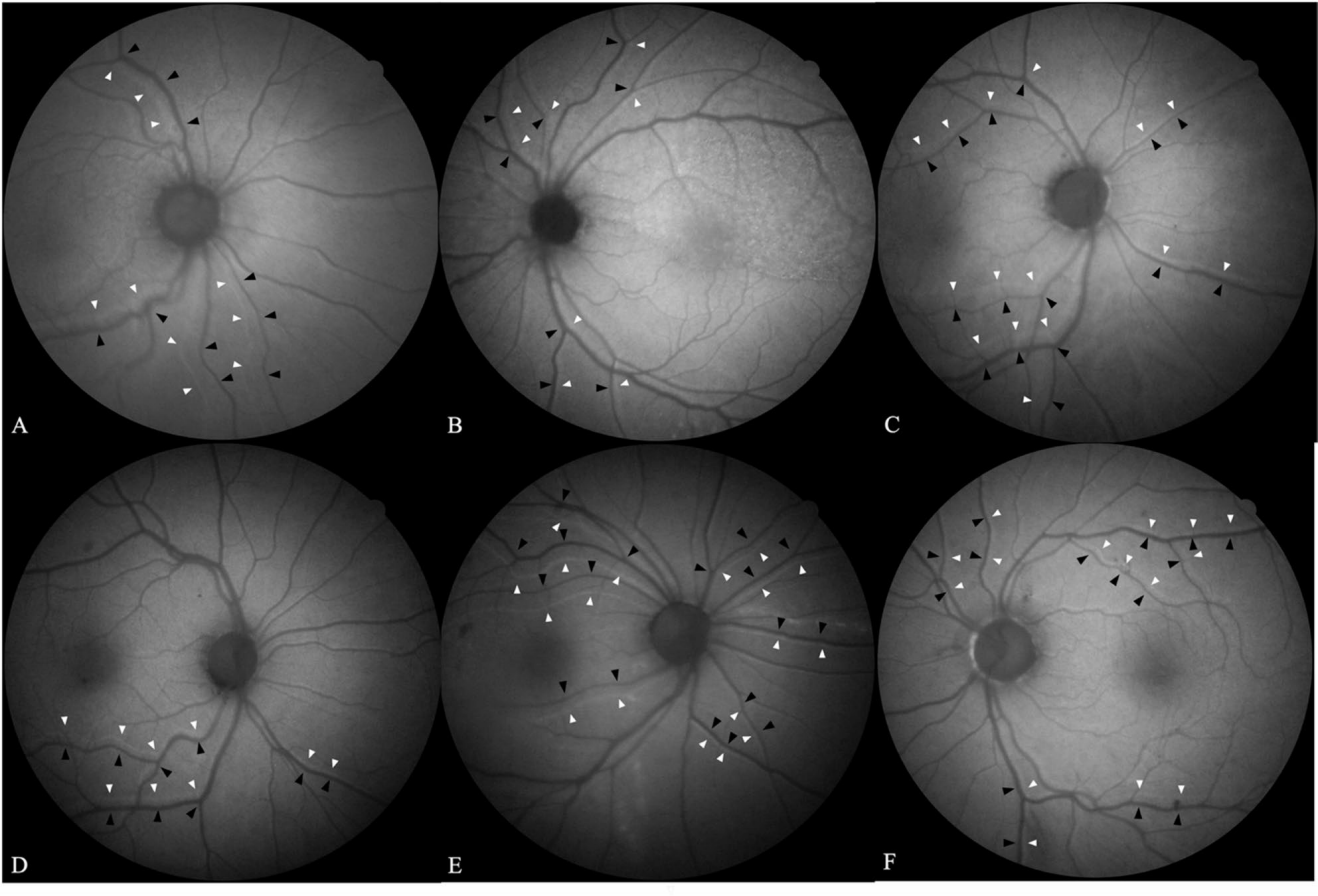
Directional and rotational features of postoperative retinal displacement on fundus autofluorescence after pars plana vitrectomy (**A**-**F**)Across all panels, white arrowheads indicate the vessels’ original (pre-detachment) positions, whereas black arrowheads indicate their postoperative (current) positions. Panels B and F represent left eyes; all other panels represent right eyes. **A**. The superotemporal arcade is displaced superiorly and the inferior arcade inferiorly; on the spherical retinal surface, superior displacement corresponds to a clockwise shift and inferior displacement to a counterclockwise shift. **B**. Superior displacement of the superotemporal arcade and inferior displacement of the inferotemporal arcade are present; the superotemporal and superonasal arcades demonstrate a counterclockwise shift, whereas the inferotemporal arcade shows a clockwise shift. A focal autofluorescence signal defect in the superior macula is consistent with an ellipsoid zone–related imaging artifact. **C**. Both temporal and nasal arcades are displaced inferiorly, with a counterclockwise shift temporally and a clockwise shift nasally. **D**. Inferotemporal and inferonasal arcades are displaced inferiorly, showing a counterclockwise shift inferotemporally and a clockwise shift inferonasally. **E**. The temporal arcades show marked superior displacement and the nasal arcades a milder superior shift; the temporal arcades demonstrate a clockwise shift and the nasal arcades a counterclockwise shift. **F**. Inferior displacement of the temporal arcades and the superior nasal arcade is observed, with a clockwise shift temporally and a counterclockwise shift in the superior nasal arcade

Postoperative retinal displacement was observed in 7 eyes (36.8%) in the PPV–SiO group and 8 eyes (61.5%) in the PPV–gas group (*p* = 0.169); however, the direction differed significantly between groups, with displacement being superior in 4 eyes (21.1%) and inferior in 3 eyes (15.8%) in the PPV–SiO group, whereas it was exclusively inferior in 8 eyes (61.5%) in the PPV–gas group (*p* = 0.016). Displacement in different directions was detected in 2 eyes (10.5%) in the PPV–SiO group and 5 eyes (38.5%) in the PPV–gas group (*p* = 0.341)(Fig. 1).

The AL was similar in eyes with and without postoperative displacement in both the PPV-SiO and PPV-gas groups (24.36 ± 1.16 vs. 24.42 ± 0.89 mm, *p* = 0.922 and 24.67 ± 2.11 vs. 24.01 ± 0.72 mm, *p* = 0.428, respectively). M-CHARTS™ change scores were generally comparable by displacement status, except for greater horizontal changes from month 1 to month 6 (*p* = 0.047) and greater mean changes from month 3 to month 6 (*p* = 0.04) in the PPV–SiO group. Detailed data are provided in Table 3.

**Table 3:** Axial length and M-CHARTS™ changes by retinal displacement status

		PPV-SiO			PPV-Gas		
		Displacement (-) *n*:12	Displacement (+) *n*:7	*p*	Displacement (-) *n*:5	Displacement (+) *n*:8	*p*
Axial Length (mm)		24,36 ± 1,16	24,42 ± 0,89	0,922*	24,67 ± 2,11	24,01 ± 0,72	0,428*
M-CHART							
MV	Δ Month 1–Month 3	0 ± 0,121	0,071 ± 0,095	0,163**†**	0 ± 0,2	-0,088 ± 0,173	0,288**†**
		0 (-0,1 − 0,08)	0 (0–0,2)		0,1 (-0,2 − 0,15)	0 (-0,1 − 0)	
	Δ Month 1–Month 6	-0,05 ± 0,211	0,057 ± 0,181	0,261**†**	0,06 ± 0,055	-0,075 ± 0,139	0,065**†**
		-0,05 (-0,18 − 0)	0 (-0,1 − 0,2)		0,1 (0–0,1)	0 (-0,2 − 0)	
	Δ Month 3–Month 6	-0,05 ± 0,117	-0,014 ± 0,122	0,467**†**	0,06 ± 0,219	0,013 ± 0,155	0,820**†**
		-0,05 (-0,1 − 0)	0 (-0,1 − 0)		0 (-0,1 − 0,25)	0 (-0,15 − 0,18)	
MH	Δ Month 1–Month 3	0,008 ± 0,211	-0,029 ± 0,221	0,758**†**	-0,08 ± 0,335	-0,025 ± 0,071	0,879**†**
		0 (-0,08 − 0,08)	0 (-0,2 − 0,2)		0 (-0,4 − 0,2)	0 (-0,1 − 0)	
	Δ Month 1–Month 6	-0,075 ± 0,148	0,043 ± 0,079	**0**,**047†**	-0,06 ± 0,451	-0,025 ± 0,117	0,758**†**
		-0,1 (-0,18 − 0)	0 (0–0,1)		-0,1 (-0,45 − 0,35)	0 (0–0)	
	Δ Month 3–Month 6	-0,083 ± 0,195	0,071 ± 0,17	0,097**†**	0,02 ± 0,192	0 ± 0,131	0,939**†**
		-0,05 (-0,2 − 0)	0 (0–0,2)		0 (-0,15 − 0,2)	0 (0–0,1)	
Mean	Δ Month 1–Month 3	0,004 ± 0,136	0,021 ± 0,135	0,829**†**	-0,13 ± 0,448	-0,056 ± 0,105	0,451**†**
		0 (-0,05 − 0,09)	0 (-0,1 − 0,2)		0,1 (-0,53 − 0,15)	0 (-0,09 − 0)	
	Δ Month 1–Month 6	-0,063 ± 0,163	0,05 ± 0,119	0,135**†**	0 ± 0,232	-0,05 ± 0,11	0,824**†**
		-0,08 (-0,2 − 0,04)	0 (-0,05 − 0,2)		-0,05 (-0,2 − 0,23)	0 (-0,14 − 0)	
	Δ Month 3–Month 6	-0,067 ± 0,098	0,029 ± 0,076	**0**,**04†**	0,13 ± 0,295	0,006 ± 0,124	0,370**†**
		-0,05 (-0,14 − 0)	0 (0–0,1)		0,1 (-0,1 − 0,38)	0 (-0,04 − 0,1)	

†Mann-Whitney U test * Independent t-test

p <0.05 was considered statistically significant

PPV: pars plana vitrectomy ; MV: metamorphopsia vertical ; MH: metamorphopsia horizontal

AL showed no significant correlation with changes in M-CHARTS™ scores (vertical, horizontal, or mean) at any interval in either tamponade group (all *p* ≥ 0.283), and detailed correlation coefficients are provided in Table 4.

**Table 4:** Pearson correlation between axial length and M-CHARTS™ change scores

			Axial Length (mm)	
			PPV-SiO	PPV-Gas
MV	Δ Month 1–Month 3	r	-0,065	-0,027
		p	0,792	0,929
	Δ Month 1–Month 6	r	-0,016	-0,019
		p	0,949	0,951
	Δ Month 3–Month 6	r	0,037	0,014
		p	0,881	0,963
MH	Δ Month 1–Month 3	r	-0,210	-0,008
		p	0,388	0,980
	Δ Month 1–Month 6	r	-0,207	0,170
		p	0,396	0,580
	Δ Month 3–Month 6	r	0,079	0,322
		p	0,748	0,283
Mean	Δ Month 1–Month 3	r	-0,195	-0,118
		p	0,423	0,700
	Δ Month 1–Month 6	r	-0,102	0,138
		p	0,678	0,652
	Δ Month 3–Month 6	r	0,099	0,266
		p	0,687	0,379

Pearson correlation test

p <0.05 was considered statistically significant

PPV: pars plana vitrectomy ; MV: metamorphopsia vertical ; MH: metamorphopsia horizontal

## Discussion

The present study included 32 eyes with macula-off RRD after phacoemulsification to determine the frequency and direction of postoperative retinal displacement on FAF after vitrectomy with SiO (*n* = 19) or 14% C₃F₈ gas (*n* = 13) and to assess associations with metamorphopsia and AL. Displacement was detected in 36.8% SiO-group eyes and 61.5% gas-group eyes. M-CHARTS metamorphopsia scores were comparable between tamponade groups and showed no significant difference over time. There was no difference in AL by displacement status in either tamponade group. It was also not correlated with changes in M-CHARTS scores. Overall, retinal displacement was common in this cohort. However, no statistically significant association was demonstrated between retinal displacement and metamorphopsia. Furthermore, AL was not significantly associated with either outcome within the range of axial lengths represented in this study.

Previous studies consistently show that macular status influences both the incidence and severity of postoperative metamorphopsia after retinal detachment repair [7, 11, 12]. However, most reports use a binary “macula-off” label that does not specify zone 1 involvement or include vascular arcades, an important limitation given the importance of zone 1 function. Zhou et al. [13] and Saleh et al. [14] identified preoperative macula-off status as an independent predictor of postoperative metamorphopsia, yet the absence of macula-off grading may partly account for variability in structure–function correlations across studies. To address this uncertainty, we classified macula-off detachment severity using the foveal involvement framework described by Klaas et al. [10] which provides a more anatomically informative stratification of central detachment patterns relevant to metamorphopsia. Notably, the distribution of foveal involvement categories was comparable between the SiO and gas groups in our cohort, indicating balanced macular detachment severity across treatment arms and thereby strengthening the internal validity of our displacement–metamorphopsia analyses relative to prior studies that did not quantify macula-off extent.

In the present cohort, BCVA was comparable between the PPV–SiO and PPV–gas groups preoperatively and at postoperative month 1, but became significantly better in the gas group at months 3 and 6. This late divergence is unlikely to be driven by differences in surgical timing or baseline macular detachment extent, given similar symptom onset-to-surgery intervals and comparable macula-off severity/foveal involvement distributions. Notably, ellipsoid zone disruption was more frequent in the SiO group at month 1, suggesting greater early photoreceptor involvement that may constrain subsequent functional recovery.In addition, early postoperative complications and day-1 intraocular pressure were higher in the SiO group, factors that could plausibly delay visual rehabilitation. Collectively, these findings suggest that tamponade selection may partially reflect baseline case complexity, while SiO-related optical and retinal factors may further contribute to less favourable BCVA at later follow-up [15]. Beyond the issue of reduced optical quality, SiO has been associated with delayed photoreceptor–RPE microstructural recovery and macular integrity.This may be due to mechanical interaction with the retinal surface and subclinical inflammatory or metabolic stress, which could manifest as slower restoration of outer retinal bands (e.g., ellipsoid zone continuity), an increased tendency for cystoid macular oedema, and, consequently, limited functional recovery [16–18]. Nevertheless, it is imperative to acknowledge that this interpretation should be regarded as biologically plausible rather than causal due to the retrospective design.

In our study, retinal displacement was observed in 36.8% of eyes treated with SiO and 61.5% of eyes treated with gas tamponade.Although the difference did not reach statistical significance, this supports the view that displacement is a frequent phenomenon that is more prominent after PPV and may be more common with gas.This is consistent with the wide incidence range reported in the literature and the influence of case selection, macular status, detachment extent, imaging timing and imaging field on detection rates [6] and comparative data show higher displacement rates after PPV than pneumatic retinopexy [19]. The most compelling discussion point in our cohort is the marked divergence in displacement direction. All displacements in the PPV-gas group were inferior, whereas the PPV-SiO group showed a bidirectional pattern. This directional signature mirrors prior FAF-based series in which gas tamponade was associated with higher displacement rates and predominantly downward displacements [20].Reports also show higher displacement with gas (71.4%) compared to SiO (22.2%), with a consistent pattern: downward with gas and upward with SiO [21]. Displacement is uncommon in some SiO-treated groups, which are often more complex.This shows that SiO may be protective but not preventative [22]. Contemporary frameworks suggest that displacement is the result of a fluid–tissue interaction during subretinal fluid redistribution and retinal re-apposition.The buoyant force and large contact interface of a gas bubble can drive a more uniform gravity-aligned vector that is typically inferior. However, the different physical behaviour of SiO, including viscosity and interfacial characteristics, may yield less frequent and more heterogeneous displacement patterns [23, 24]. Postoperative management can modify displacement risk and magnitude.Evidence shows positioning strategies reduce the rate and amplitude of displacement [25]. Greater displacement amplitude correlates with higher distortion scores.This explains why directional/quantitative FAF findings may be clinically meaningful. Broad syntheses emphasising tamponade choice indicate that gas is associated with a higher displacement risk than SiO [26, 27].

To the best of our knowledge, most studies of retinal displacement after retinal detachment repair describe a single predominant direction per eye, typically reported as a fixed, eye-level variable [20, 21, 27–30]. In our study, we additionally identified a mixed direction phenotype within the same eye that could be interpreted as multi-vector or rotational displacement, and this pattern was more frequent in the gas group than in the SiO group (38.5% vs. 10.5%), which suggests that in some eyes the postoperative mechanical environment is better described by a spatially heterogeneous shear field rather than a single uniform displacement vector.Clinically, two nonexclusive mechanisms may explain this finding, because a broad detachment with asymmetric residual subretinal fluid pockets can create multiple local drainage pathways, and postoperative variability in head posture and ocular versions can change quadrant-specific tamponade coverage over time, particularly as the gas bubble shrinks and interfacial effects alter the distribution of contact pressure.Simulation and physics-based work supports the idea that bulk flow of subretinal fluid away from the tamponade along gravity can stretch the retina and generate displacement, and that displacement magnitude depends on subretinal fluid thickness and the tamponade contact area and pressure, which are greater with larger gas fills and differ between surgical models and tamponades [24, 31] while computational fluid dynamics studies also show that tamponade-retina contact can vary substantially with fill percentage and posture, providing a plausible pathway for time-varying regional coverage that could redirect fluid vectors in different macular sectors [32]. In these conditions, it may be more informative to interpret the FAF phenotype in a broader way, considering other outcomes driven by subretinal fluid flow and tissue stretch [27, 29]. The framework predicts localised strain and photoreceptor perturbation.Recent data support the early postoperative signs of displacement, reinforcing the need to characterise complex phenotypes beyond FAF vessel imprints [33]. OCT is central to interpreting structure and function.It can quantify the outer retinal fold burden early after surgery, and early fold density can predict metamorphopsia.Enhanced depth or ultra-high resolution OCT studies link foveal displacement and outer layer disorganisation to metamorphopsia severity [34, 35]. For this reason, OCT acquisition sampling meridians parallel and perpendicular to the observed FAF vectors may increase the likelihood of capturing the microstructural correlates of the underlying heterogeneous shear field and clarifying how this phenotype relates to postoperative visual symptoms.

Changes in M-CHARTS according to displacement status were highly comparable. Horizontal metamorphopsia in displacement-positive cases changed more between 3 and 6 months, and the mean score change between 3 and 6 months was more pronounced in these cases, suggests two important considerations.First, metamorphopsia may not be driven solely by a binary displacement status but rather by more nuanced factors such as the magnitude of displacement, microstructural alterations including outer retinal folds, and subtle changes in the photoreceptor bands.Accordingly, in a small sample, these relationships may be detected only intermittently across time intervals [35, 36]. Directional heterogeneity in the SiO group can lead to variable patterns of tangential stress across the retina, particularly the macula, which can selectively influence the horizontal and mean components of the M-CHARTS. Prospective evidence suggests that retinal folds and photoreceptor layer alterations may be early markers associated with retinal displacement [33, 36].

There was no difference in AL between eyes with and without postoperative retinal displacement in either group. Furthermore, no significant association was found between AL and changes in M-CHARTS scores during follow-up. These findings suggest that, within the AL range represented in this cohort, AL alone was not a major determinant of postoperative functional distortion. However, this result should be interpreted with caution because the sample size was small and the AL distribution was relatively narrow. Although a small number of eyes in our cohort had an AL greater than 26 mm, such eyes were underrepresented. This is important when interpreting our findings in relation to the FAF-based study by Mancini et al. [8], which suggested that longer AL, particularly above 26 mm, may be associated with a higher risk of postoperative retinal displacement. Therefore, the limited representation of highly elongated eyes in our cohort may have reduced our ability to detect such an association. From a mechanistic perspective, this interpretation remains compatible with the model proposed by Mancini et al. [8], in which AL may indirectly influence the risk of displacement and metamorphopsia through its effect on globe geometry, tamponade contact height, and pressure distribution across the retinal surface. In that framework, longer AL may alter the dynamics of retinal reapposition and may also increase the detectability of displacement traces on two-dimensional FAF imaging. Accordingly, our findings should be interpreted as showing no significant relationship within the AL spectrum represented in the present study, rather than as definitively excluding a possible association in eyes with greater AL.

The main limitations of this study include its retrospective design and relatively small sample size.Because of the retrospective design, standardised data regarding pre-phacoemulsification retinal findings and prophylactic management were not available for all eyes.In addition, tamponade choice was not protocol-driven but left to the operating surgeon’s intraoperative judgement, which may have introduced selection bias between the SiO and gas groups.

In conclusion, retinal displacement was frequently observed in this series and occurred more often after gas tamponade, exhibiting a predominantly uniform inferior-direction pattern, whereas SiO tamponade was associated with a more heterogeneous directional profile.AL was not associated with retinal displacement or metamorphopsia in this cohort and postoperative visual distortion may be better explained by early microstructural strain signatures such as outer retinal folds detectable on OCT rather than by binary displacement status alone.

## Data Availability

The datasets used and/or analysed during the current study are not publicly available due to patient confidentiality and institutional restrictions but are available from the corresponding author on reasonable request.
